# The clinicopathological features of breast cancer in Peutz-Jeghers syndrome: results from an international survey

**DOI:** 10.1007/s10689-025-00469-5

**Published:** 2025-05-03

**Authors:** Elizabeth Loehrer, Anja Wagner, Massiah Bahar, F. Rubab Ramzan, Anne Marie Jelsig, Anne Goverde, Monique van Leerdam, Susanne E. Korsse, Evelien Dekker, Manon C. W. Spaander, John Gásdal Karstensen, Veronica Zuber, Finlay Macrae, Andrew Latchford

**Affiliations:** 1 Department of Clinical Genetics, Erasmus MC Cancer Institute, Rotterdam, Netherlands; 2 St Mark’s Hospital, Harrow, UK; 3 Royal Melbourne Hospital, Melbourne, Australia; 4 Department of Clinical Genetics, Copenhagen University Hospital - Rigshospitalet, Copenhagen, Denmark; 5 Netherlands Cancer Institute, Amsterdam, Netherlands; 6 Frisius Medical Center, Leeuwarden, The Netherlands; 7 Amsterdam UMC, Amsterdam, Netherlands; 8 Department of Gastroenterology & Hepatology, Erasmus University Medical Center, Erasmus MC Cancer Institute, Rotterdam, the Netherlands; 9 Breast Surgery Unit, University and Research Hospital San Raffaele, Milan, Italy; 10 Department of Surgery and Cancer, Faculty of Medicine, Imperial College, London, SW7 2AX UK; 11 Danish Polyposis Register, Gastro Unit, Copenhagen University Hospital - Amager and Hvidovre, Hvidovre, Denmark; 12 Department of Clinical Medicine, University of Copenhagen, Copenhagen, Denmark

**Keywords:** Genetic cancer predisposition, Peutz-Jeghers syndrome, Breast cancer, Hereditary cancer syndromes, Germline pathogenic variants

## Abstract

Background: Female patients with Peutz-Jeghers syndrome (PJS) have an increased risk of breast cancer (BrCa), and surveillance is recommended. However, clinicopathological features of their tumors and prognosis are lacking. To facilitate more precise future guideline development, we evaluated these data. Methods: We conducted an international survey for InSiGHT members to collect retrospective data on PJS patients with diagnosed breast cancer. Results: We received 23 responses, including three centers with data on BrCa patients. All reported BrCa patients were female. In total, the cohort comprised 27 patients with 34 BrCa (five bilateral synchronous, one bilateral metachronous, and one metachronous unilateral tumours). The median age at first cancer diagnosis was 45 years (range 26–67). Most cancers were ductal carcinoma, either invasive (13) or in situ (DCIS; 19). TNM staging for invasive cancer was available in thirteen cases, of which nine were T1N0M0. Among tumors with histological reports, 14/15 were oestrogen receptor positive, 8/15 were progesterone receptor positive, and 4/15 were HER2 positive. There were no triple negative breast cancers. Twenty-five patients had follow-up data, comprising 229 patient years. Eleven patients had died of any cause during follow-up. Survival at 5 years was 73%. Conclusion: Overall, breast cancers that occur in this PJS population seem to have favorable characteristics and prognosis. These data will help inform discussions about risk management in patients with PJS. Further research is needed to better understand lifetime risk, the optimal surveillance modality and its outcomes.

## Introduction

Peutz-Jeghers syndrome (PJS) is a rare autosomal dominant inherited disorder, caused by a constitutional pathogenic variant (PV) in the tumour suppressor gene *STK11* (also known as *LKB1*) [1, 2] and clinically characterized by the development of Peutz-Jegher type hamartomatous polyps in the gastrointestinal tract, mucocutaneous pigmentation [3–5] The estimated prevalence of PJS is most consistently reported to be somewhere between 1 in 50,000 to 1 in 200,000, with a wider range of estimates from some studies [5–7]. Patients with PJS have an increased cancer risk as well as an increased risk of cancer-related mortality [8–16]. The absolute risk of developing cancer among PJS patients is difficult to estimate given the rarity of this condition and the nature of historical studies which are likely to be subject to significant ascertainment bias [5, 17]. Women with PJS are reported to have a 19–54% lifetime risk of developing breast cancer [8, 9]. This wide variation in quoted risk probably reflects ascertainment bias and small number of absolute cases in the available data (ranging from 1 to 17 reported breast cancer cases per previously published case series) [8–14, 16, 18]. Nonetheless, the breast cancer risk in women with PJS appears consistently elevated compared to general female population risk between 12 and 14% observed in Western countries [19–21]. Due to these striking numbers, the fifth edition of the World Health Organization classification of tumours of the breast (2019) now includes PJS as a genetic tumour syndrome of breast cancer [22], in line with other PVs at high or moderate risk for breast cancer, including established PVs in *BRCA1*,* BRCA2*, *CDH1*,* PALB2*, *ATM*, *CHEK2*, *PTEN*, and *TP53* [23, 24]. 

Because of their estimated high lifetime risk of breast cancer [17, 25–27], women with PJS are recommended to start surveillance with annual breast MRI and clinical breast exams every 6–12 months by age 25 or 30 [17, 27, 28]. As the breast tumor biology, clinical characteristics and prognosis among women with PJS have not been characterized in the same way as breast cancers in persons with other high risk PVs [29], routine risk reducing mastectomy is currently not recommended [17]. A few case reports have documented the occurrence of both synchronous and metachronous bilateral breast cancers in women with PJS [18, 30–33]. A few recent case reports and case series have reported on histological subtypes of the breast tumors occurring in women with PJS, and most have reported ER + tumors [34, 35], though one has reported a triple negative breast cancer (TNBC) that demonstrated loss of the normal homologous *STK11* allele in the tumor [36]. Overall, information about presentation and prognosis of breast cancer in PJS is limited, and this information is essential to inform counseling and to help future development of surveillance and treatment guidelines.

To begin to address this gap, we have conducted an international survey to provide a descriptive epidemiologic assessment of the clinical and pathological characteristics of breast cancers among PJS patients.

## Methods

We performed a retrospective survey to assess information about the histological classification, receptor status, and clinical care of breast tumors among PJS patients. PJS patients were eligible for inclusion if they had histologically confirmed diagnoses of breast tumor (invasive cancers and/or ductal carcinoma in situ).

### Study populations

The International Society for Gastrointestinal Hereditary Tumors (InSiGHT) is a scientific organization for researchers, clinicians and other healthcare professionals focused on research and clinical care into hereditary conditions that predispose to gastrointestinal tumors, including PJS [37, 38]. We sent the survey to InSiGHT members to enquire if they had had a case of PJS and breast cancer. For those that did, we collected retrospective information about breast cancers that had occurred in their PJS populations.

Prior to distributing the survey, local institutional Research and Development approval was received and each center contributing data obtained local institutional approval to share anonymized data.

### Data visualization and analysis

Survey responses and anonymized data were collated and information about patient follow-up and confirmation of vital status was checked by clinicians at participating sites in the third quarter of 2023. Descriptive statistics and data visualization of the survey results, including a Kaplan-Meier plot of overall survival, were generated using IBM SPSS Statistics for Windows, Version: 28.0.1.0 (Amronk, NY: IBM Corp). Due to the small numbers of cases and considerable missing data, we did not perform statistical tests to compare the data across strata, and report only descriptive statistics here. Given the retrospective nature and lack of coordinated, prospectively collated surveillance outcomes, this study is not intended to address cumulative risk of BrCa development in this population.

## Results

All registered InSiGHT members were contacted and 23 responses were received. Three centers had cases of PJS and breast cancer and one center had detailed breast surveillance data but no cases of breast cancer [15]. All cases were either confirmed carriers or obligate carriers of likely pathogenic or pathogenic germline variant in *STK11* (Table 1).There were 34 breast cancers in 27 patients (including five bilateral synchronous tumors, one case with bilateral metachronous tumors, and one metachronous unilateral tumor). Two additional patients were diagnosed with benign breast lesions, and they were excluded from further analysis. All reported breast cancer patients were female (Table 2). The median age at first cancer diagnosis was 45 years (range 26–67). About one third of DCIS tumors and invasive ductal carcinoma tumors were diagnosed in women age < 40 years.

**Table 1:** Genetic information of Peutz-Jeghers patients who had breast cancer diagnoses (*N* = 27 patients)

Patient number	STK11 variant
1	c.370 A > T
2	c. 752 G > A
3	Not tested
4	c.735-1 G > A*
5	c.Del Ex 1
6	c.921 − 12 G > A*
7	c.921 − 12 G > A
8	c.991 dupC
9	c.197 dupT
10	c.290 + 1 G > C
11	c.908 T > A
12	c.664 delA
13	c.396 C > A
14	c.368 delA
15	c.843 delG
16	c.843 delG
17	c.454 C > T
18	c.454 C > T
19	c.454 C > T
21	c.815–816 insA
22	c.Ex7 R304W
23	c.Del ex 3–4
24	c.Del ex 3–10
25	c.256 C > T
26	c.250 A > T
27	c.232 A > T
28	c.1-? 464 + del

*mutation detected in first degree relative

**Table 2:** Characteristics of Peutz-Jeghers patients who had breast cancer diagnoses (*N* = 27 patients)

Female	27 (100%)
Age at first breast cancer diagnosis, years	45 [26–67]
Death, all cause	9 (33.3%)
Follow-up time, years^*^	9.5 [0–29]

Values reported are absolute numbers(% of the population with information available) or median[interquartile range]

^*^ Follow-up time was missing for 5 patients

The reported tumors were diagnosed between 1944 and 2020. Given the historical nature of this survey, several cases predate breast cancer surveillance efforts in their countries. Among those who did take part in surveillance, we do not have detailed, country-specific information about the recommendations for modality or frequency of surveillance at the time of diagnosis for the individual cases; guidelines have been updated during the retrospective study period. Information about (prior) breast cancer surveillance or tumor detection were available for 14 patients, and the results are presented in Table 3. Among the 9 patients with information about tumor detection, 5 tumors were reported to be detected at surveillance, including 2 tumors detected at first surveillance, and 4 tumors were detected as a palpable mass after a previous negative mammogram. We caution against drawing conclusions about surveillance based on these data since the information was incomplete and inconsistently reported.

**Table 3:** Surveillance and tumor detection

Patient number	Surveillance-detected tumor	Surveillance history	Comments
1	1	No prior surveillance	Tumor detected at first surveillance
2	0	Prior surveillance	Previous mammogram was 7 months earlier (BI-RADS 2)
3	0	No prior surveillance	Tumor detected as palpable mass
4	1	Yearly surveillance	
5	0	Prior surveillance	Tumor detected as palpable mass; Last surveillance was 6 years earlier
6	1	Prior surveillance	Previous mammogram was 3 years earlier
7	1	No prior surveillance	Tumor detected at first surveillance
8	0		Tumor was not surveillance-detected, and we have no information about prior surveillance
9	0	Prior surveillance	Tumor detected as palpable mass; Previous mammogram was 7 months earlier (BI-RADS 2)
10	1	Prior surveillance	Previous mammogram was 14 months earlier
11	0	Prior surveillance	Tumor detected as palpable mass; Last surveillance was 8 months earlier
13	1		Tumor was surveillance-detected, but we have no information about prior surveillance
27		Yearly surveillance	Patient had prior surveillance (mammogram and MRI), but we have no information if tumor was surveillance-detected
28		Yearly surveillance	Patient had prior surveillance (ultrasound and MRI), but we have no information if tumor was surveillance-detected

### Tumor characteristics

An overview of the tumor characteristics is presented in Table 4. Half of the cancers were ductal carcinoma in situ (DCIS, *N* = 17) followed by invasive ductal carcinoma (ICD, *N* = 14), intracystic papillary carcinoma (*N* = 1), invasive mixed (*N* = 1), and unspecified invasive tumor (*N* = 1). For three DCIS, information on grade was missing (17.6%). Of the DCIS with grade available (*N* = 11), six DCIS (42.9%) were grade 3, and seven DCIS (50%) were grade 2 at the time of diagnosis. Among the invasive tumors with TNM staging available (*N* = 14), nine had tumors with stage T1N0M0, two had T2N0M0, and three were lymph node positive. No patient presented with metastatic disease. Among the invasive tumors with information on receptor status available(*N* = 11/17), all eleven were ER+, four were PR + and three were HER+. Additionally, among the four DCIS with receptor status available, three were ER+, four were PR+, and one was HER2+. There were no triple negative breast cancers.

**Table 4:** Breast tumor characteristics at the time of diagnosis

Patient	Tumor	Breast	Age	Grade	TNM	ER+	PR+	HER+	Surgery	Chemo	Radiation	Endocrine	Tumor type
1	1		34	2	T0N0M0				Lumpectomy	0	1		DCIS
8	1		61	2	T0N0M0								DCIS
11	1		26	3	T0N0M0				Bilateral ablation	1	0	1	DCIS
12	1		33	3	T0N0M0								DCIS
13	1		47			1	1	0	Bilateral mastectomy				DCIS
14	1	L	61	3	T0N0M0				Mastectomy				DCIS
14	2	R	61		T0N0M0				Mastectomy				DCIS
15	1	L	34	2	T0N0M0				Mastectomy				DCIS
17	1	R	36	3	T0N0M0	1	1	0	Bilateral mastectomy				DCIS
17	1	L	36	2	T0N0M0	0	0	Equiv					DCIS
22	1	R	62	2	T0N0M0				Mastectomy				DCIS
23	1	R	45	2	T0N0M0				Lumpectomy				DCIS
24	1	R	41										DCIS
24	1	L	41										DCIS
26	1	R	49	3	T0N0M0	1	1	0	Mastectomy				DCIS
26	1	L	49	1	T0N0M0				Lumpectomy				DCIS
27	1	L	45	2	T0N0M0				Bilateral mastectomy				DCIS
28	1	R	28	3	T0N0M0	0	0	1	Bilateral mastectomy				DCIS
2	1		50		T1N0M0	1	1	0	Lumpectomy	1	1	1	IDC
3	1		34	3	T2N0M0	1	1	0					IDC
5	1		49		T1N0M0	1	1	0	Lumpectomy	0	1	0	IDC
6	1		53		T1N0M0	1	0	0	Lumpectomy	0	1	0	IDC
7	1		61		T1N0M0								IDC
9	1		47						Lumpectomy + ablation	0	0	0	IDC
10	1		41		T1N0M0	1	0	0	Ablation	1	0	0	IDC
11	1		26		T1N1M0	1	1	0	Bilateral ablation	1	0	1	IDC
14			51										IDC
16	1		36		TxN2				Mastectomy	0	1		IDC
18	1	R	35		T1N0M0	1	0	0	Bilateral Mastectomy	1	1	1	IDC
18	1	L	35		T2N1M0	1	0	1					IDC
19	1	R	67		T1N0M0	1	0	0	Lumpectomy	1	0	1	IDC
25	1	R	31		T1N0M0	1	0	1	Bilateral mastectomy	1	1	1	IDC
21	1	R	46		T1N0M0	1	0	1	Lumpectomy and bilateral mammoplasty	0	1	1	Invasive mixed
15	2	L	44						Left lumpectomy and right mastectomy				Invasive papillary
4	1		55		T2N0M0				Lumpectomy	1	1		Unspecified invasive

In the columns ER+, PR+, HER2, Chemo, Radiation, and Endocrine, a 1 denotes “yes”, and a 0 denotes “no”. All empty cells denote the data is not known. DCIS: ductal carcinoma in situ; IDC: invasive ductal carcinoma

### Treatment

Most patients were treated with some form of surgery for their first breast tumor diagnosis. Six patients underwent bilateral mastectomy, while five patients underwent a unilateral mastectomy, of whom two patients were later treated for a second breast tumor with mastectomy of the other breast. Ten women were treated with lumpectomy. Among the invasive tumors with information about additional treatment available (*N* = 15/17), eight women were treated with chemotherapy, nine women were treated with radiotherapy, and seven women were treated with endocrine therapy (Table 3).

### Follow-up

**Fig. 1:**
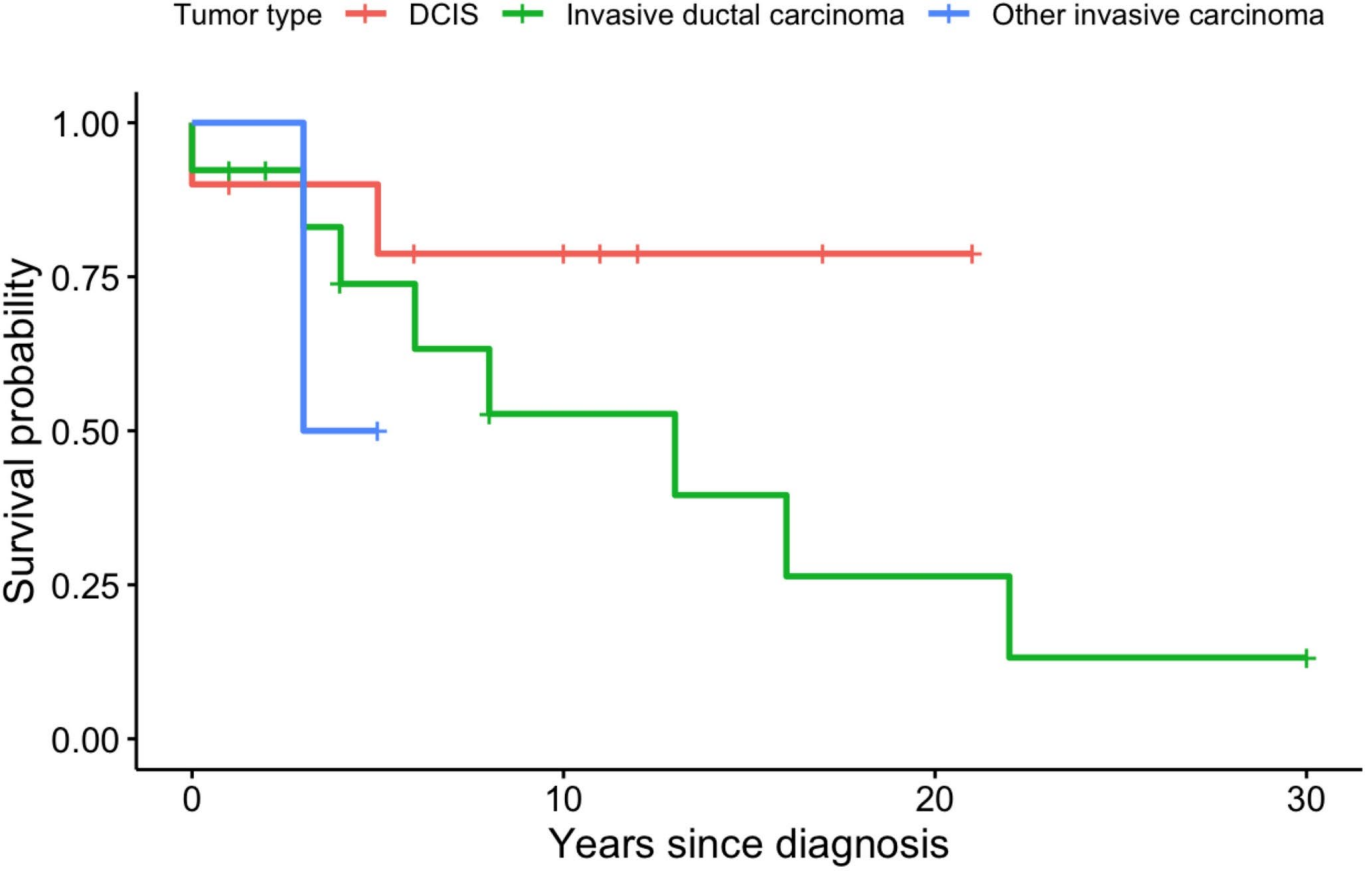
Kaplan-Meier curve denoting overall survival by first tumor histology

Twenty-five patients had information on follow-up time available, with a total of 229 person-years of follow-up. Eleven patients are reported to have passed away during follow-up. Among those who died, median age of death was 56 years (range 39–75). Six of the eleven women passed away within 5 years of the breast cancer diagnosis (5-year cumulative survival 73.0%), though we do not have information on cause of death to confirm that these were cancer related. For two patients, follow-up time information was missing, but among the patients who were still known to be alive at the time of the survey (*N* = 14), there was a median of nine years follow-up (range 1–30 years) since the diagnosis of their first tumor. When stratified by histological subtype (i.e. DCIS, invasive ductal carcinoma, or other invasive carcinoma), we see that DCIS patients seemingly had no deaths reported after 5 years, and 50% of the DCIS patients had ≥ 10 years of follow-up (Fig. 1). For patients with invasive ductal carcinoma, the median overall survival time was estimated to be 13 years after diagnosis of the first tumor, though the small sample size leads to uncertainty in this estimate (95% CI 3.5–22.5 years). Of the other invasive cancers diagnosed, the patient with an unknown invasive breast carcinoma passed away three years after her diagnosis, while the other patient was censored after five years. Of note, these survival curves do not account for competing risks, such as new primary diagnoses, or other cancer diagnoses, nor do they account for year of diagnosis, staging and treatment of the primary tumor.

## Discussion

This is the first study evaluating the breast tumor characteristics in an internationalpopulation of PJS patients. All breast tumors reported in our survey occurred in women. Half of the tumors reported were DCIS, and among the cases with information about hormone receptor status, we had no reports of triple negative breast cancers.

In the absence of precise risk characterization, women with known *STK11* PVs and likely PVs [39] are being treated equivalently to *BRCA1* and *BRCA2* PGV carriers. Similar to *BRCA1* and *BRCA2* PV carriers [40], breast cancers do occur at a young age in women with PJS. Previous studies estimate the median age of breast cancer diagnosis in PJS to be 37 years [29]. In our survey patient population, the median age at first cancer diagnosis was 45 years old with a range from 26 to 67 years old. Several patients were diagnosed with synchronous or metachronous bilateral breast tumors, and two women experienced recurrent tumors during their follow-up. This is consistent with numerous other case reports of bilateral breast tumors among women PJS [18, 30–33], and reinforces the overall increased risk of developing breast tumors in this population. There were no cases of men with breast cancer, confirming data in previous case studies [8, 9, 11, 34, 41]. 

Compared to women with *BRCA1* and *BRCA2* PVs, our data indicate that women with PJS have more favorable tumor characteristics. One clear comparative indication is the presence of triple negative breast cancers (TNBC) (i.e. tumors that do not have receptors for estrogen or progesterone and do not produce HER2 protein), which carry the worse prognosis among breast cancer subtypes [42, 43]. TNBCs make up 10–20% of all breast cancer diagnoses. Women with *BRCA1*, and to a lesser extent *BRCA2* PVs, have an even higher risk of TNBCs compared to the general population [42, 44, 45]. We are aware of one case study in the literature in which a woman with a germline PV in *STK11* was diagnosed with a TNBC, in which the tumor showed loss of homologous normal allele [36]. In this study sample, we observed no TNBC’s in PJS patients. All invasive tumors in our study for which immunohistochemistry was available were hormone receptor positive, in line with several previous case reports of breast tumors in women with PV in *STK11* [34, 35]. Due to the limited sample size, this result should be interpreted with caution.

Notably, half of the tumors diagnosed in this study population (50%) were DCIS. Actually, DCIS represent a pre-cancer that is not invasive at the time of detection, and usually DCIS have a very good prognosis [46]. About half of high-grade DCIS can progress to invasive breast cancer within five to ten years, if left untreated [47], and about 5% of treated DCIS will recur as an invasive breast tumor [48–51]. Current evidence suggests that low-grade DCIS tumors take decades to progress to invasive tumors, if they ever do [47, 52, 53]. In general population, DCIS make up between 13 and 25% of all screen-detected breast cancers, and DCIS is equally as prevalent in patients who carry *BRCA* PVs as in high familial-risk women who are non-carriers, but occurs at an earlier age [54]. The increased incidence of DCIS diagnosis after the commencement of population-based screening has fueled discussions about overdiagnosis and overtreatment of DCIS in women with average lifetime risk of breast cancer [46, 55, 56], and at least one ongoing trial in the general population is specifically studying active surveillance without treatment for women with low grade DCIS [57]. For now, the absence of informative and effective risk stratification precludes a more tailored approach for DCIS, particularly in women with a lifetime high risk of breast cancer.

The overall favorable tumor characteristics among our study population indicate a need for reflection on current clinical care and counseling for women with PJS. We note some differences in standard of care across the countries included in this survey. In the UK and Australia, women with PJS are still counseled on risk reducing bilateral mastectomy, despite recent guidelines that advise against this [17, 28, 58]. In the Netherlands, prophylactic mastectomy is generally not advocated to women with PJS in the absence of clear evidence of the clinical benefit [17]. This survey indicates that the tumors occurring in women with PJS seem to be slower growing, less likely to recur, and have better prognosis than tumors that are reported in other high-risk breast cancer groups, like *BRCA1* and *BRCA2* PV carriers. Based on this, we consider that risk reducing bilateral mastectomy may be overtreatment in this group and should not be recommended routinely.

Given the receptor status of the tumors, patients may seek additional advice on exogenous hormone use (e.g. oral contraceptives or hormone replacement therapy(HRT)). Based on the limited literature from women with high familial risk or PV in *BRCA1/2*, exogenous hormones are not contraindicated in unaffected women, but patients with previous breast cancer (especially hormone receptor positive cancer) are advised against exogenous hormone use [59–63]. For the PJS population, a potential increased risk of cervical cancer is also relevant for HRT use [5, 27, 64, 65]. Numerous international guidelines suggest endocrine therapy for breast cancer prevention in women at high risk [66–69]. All of the tumors that were tested for receptor status in our survey were ER+, suggesting preventative selective estrogen receptor modulators may be effective breast cancer prevention among PJS patients, as has been demonstrated among other patients at high risk for ER + tumors [69–71]. However, our limited understanding of the pathophysiology of breast cancer among PJS, the generally bad acceptance and adherence to chemoprevention [72, 73], and the potential for SERM to increase risk of other cancers (e.g. endometrial cancer) [74, 75], it is difficult to determine the net benefits and harms of chemoprevention in this patient group at this moment [76, 77]. 

Information about (prior) breast cancer surveillance was collected as part of the survey, but given the historical nature of this survey and the large timespan of the diagnoses (1944–2020), characterizing the role of surveillance is difficult. Several cases occurred prior to commencement of any imaging-based surveillance, and recommendations regarding starting age, modality (i.e. clinical breast exams, mammograms, and/or breast MRI) and frequency of surveillance for PJS patients (and the general public) has differed across countries and across time. From the survey responses, only five of nine tumors were surveillance detected from mammography, and four tumors presented as a palpable mass after a previously negative mammogram. The current European PJS surveillance guideline recommends utilizing contrast-enhanced breast MRI starting from age 25–30, and then some combination of annual mammography, tomosynthesis, or ultrasound in combination with breast-MRI annually up to and including age 70 years [17]. We do not have any reported information about what surveillance recommendations the patients in our survey received and only limited information about surveillance with modalities other than mammography. To date, there are no prospective data comparing the clinical efficacy and utility of various (combinations of) imaging modalities in PJS. Most of the tumors identified in this survey appear to be prognostically favorable. As such, future studies should consider the positive and negative predictive values, cost, and harm-benefit ratios of mammography, tomosynthesis and MRI-based surveillance among PJS patients and aim to calibrate the starting age and frequency of surveillance to best suit this population.

This study represents the first international survey of clinical pathological features of breast cancer among PJS patients. Though our survey includes a small sample size and is based on retrospective data collection, it provides a first profile of breast cancer in this patient population. Anecdotally, in the process of collecting this survey data, we noted that at both a national and institutional level, many InSIGHT members reported that there was no registry for patients with PJS to identify cases of breast cancer or for co-ordination of patient care more generally. In the absence of such registries, we cannot rule out that our reported cases are not subject to ascertainment bias. The small number of reported breast cancer cases may also indicate that ascertainment bias in historic data sets has led to an overestimation of breast cancer risk in this population.

To achieve an accurate characterization of breast cancer risk in PJS, we need an accurate count of all women with PJS, but we also need more precise capture of how many women with PJS undergo risk reducing bilateral mastectomy, capture other incident cancers, and capture of cause-specific mortality. Further, additional evidence is needed to determine if non-carrier family members of patients with PJS also experience an increased risk of breast cancer or other cancers due to family history [78–80]. Most importantly, there is a need for continued and reinforced collaboration between clinical genetics and oncology departments to follow patients from genetic diagnosis through cancer care in order to accurately characterize the clinical course for these patients and ensure optimal surveillance and care. Next steps should determine whether counseling and surveillance guidelines for breast cancer should be updated for women with PJS and ideally unified across countries.

## Data Availability

The available anonymized clinical data is provided within the manuscript.
